# Allelic Diversity at Abiotic Stress Responsive Genes in Relationship to Ecological Drought Indices for Cultivated Tepary Bean, *Phaseolus acutifolius* A. Gray, and Its Wild Relatives

**DOI:** 10.3390/genes12040556

**Published:** 2021-04-12

**Authors:** María A. Buitrago-Bitar, Andrés J. Cortés, Felipe López-Hernández, Jorge M. Londoño-Caicedo, Jaime E. Muñoz-Florez, L. Carmenza Muñoz, Matthew Wohlgemuth Blair

**Affiliations:** 1 Facultad de Ciencias Agropecuarias, Universidad Nacional de Colombia, Palmira 763533, Colombia; mabuitragob@unal.edu.co (M.A.B.-B.); jmlondono@uniquindio.edu.co (J.M.L.-C.); jemunozf@unal.edu.co (J.E.M.-F.); lcmunozf@unal.edu.co (L.C.M.); 2 Grupo de Investigación en Biodiversidad y Biotecnología (GIBUQ), Universidad del Quindío, Armenia 630002, Colombia; 3 Corporación Colombiana de Investigación Agropecuaria AGROSAVIA, C.I. La Selva, Km 7 vía Rionegro—Las Palmas, Rionegro 054048, Colombia; acortes@corpoica.org.co (A.J.C.); luflopezhe@unal.edu.co (F.L.-H.); 4 Facultad de Ciencias Agrarias—Departamento de Ciencias Forestales, Universidad Nacional de Colombia—Sede Medellín, Medellín 050034, Colombia; 5 Department of Agricultural & Environmental Sciences, Tennessee State University, Nashville, TN 37209, USA

**Keywords:** abscisic acid-, stress-, and ripening-induced (*Asr*) gene, candidate gene approach, climate adaptation, dehydration responsive element binding (*Dreb*) gene, drought tolerance, environmental indices, LRR receptor-like serine/threonine-protein kinase *ERECTA*-encoding gene, *Phaseolus parvifolius* Freytag, Thornthwaite’s potential evapotranspiration (PET) model

## Abstract

Some of the major impacts of climate change are expected in regions where drought stress is already an issue. Grain legumes are generally drought susceptible. However, tepary bean and its wild relatives within *Phaseolus acutifolius* or *P. parvifolius* are from arid areas between Mexico and the United States. Therefore, we hypothesize that these bean accessions have diversity signals indicative of adaptation to drought at key candidate genes such as: *Asr2*, *Dreb2B*, and *ERECTA*. By sequencing alleles of these genes and comparing to estimates of drought tolerance indices from climate data for the collection site of geo-referenced, tepary bean accessions, we determined the genotype x environmental association (GEA) of each gene. Diversity analysis found that cultivated and wild *P. acutifolius* were intermingled with var. *tenuifolius* and *P. parvifolius*, signifying that allele diversity was ample in the wild and cultivated clade over a broad sense (*sensu lato*) evaluation. Genes *Dreb2B* and *ERECTA* harbored signatures of directional selection, represented by six SNPs correlated with the environmental drought indices. This suggests that wild tepary bean is a reservoir of novel alleles at genes for drought tolerance, as expected for a species that originated in arid environments. Our study corroborated that candidate gene approach was effective for marker validation across a broad genetic base of wild tepary accessions.

## 1. Introduction

Identifying and characterizing novel sources of tolerance to abiotic stresses is among the most pressing requirements for coping with the effects of climate change on crop production [1]. Climate modeling forecasts that increased drought alone will jeopardize global crop production by over 10% sooner than 2050 [2], substantially worsening global malnutrition in the most vulnerable areas, which are also the poorest. Therefore, species and landraces locally adapted to dry environments [3] will prove keys to meet future demands. These exotic germplasm sources compared to those we use today, may either confer the necessary alleles to make cultivars more tolerant via backcrossing and genome editing [4], or they could stand as novel crop alternatives by themselves [5] if they convey local market preferences required to feed people in semi-arid regions.

Legumes from temperate and tropical regions (the true beans and peas) are known for high dietary protein and micronutrient contents, but are generally drought susceptible [6], while some semi-tropical and Mediterranean species are more tolerant [7,8]. Among the five cultivated species of the well-liked bean genus *Phaseolus*, the most drought tolerant as a whole is the tepary bean *s.s.* (*Phaseolus acutifolius* A. Gray), an annual autogamous species from northwest Mexico, likely domesticated one or twice near the arid border with the USA [9,10,11], and with a strong preference for hot and dry environments [12,13,14]. Generally, tepary beans are considered more drought tolerant than common bean (*P. vulgaris* L.) because of their origin in dryland environments [15] and have been recommended for breeding across species boundaries. Wild relatives of tepary bean such as *P. parvifolius* may be even more drought tolerant given their desert collection sites; yet this remains poorly studied as some tepary beans are ephemeral plants that appear after desert rains at the borders of dry upland forests [16].

Even though tepary bean has limited relevance as a modern crop, it is still a possible donor of alleles for tolerance [16,17,18]. Previous works have already explored this potential for heat tolerance, but it is less well explored for drought [17]. Despite this, common bean (*P. vulgaris* L.) has been backcrossed with tepary bean, yielding successful drought and disease resistance [19,20,21] despite their phylogenetic distance [6,22,23] and low levels of inter-specific introgression [16]. Yet, these efforts have been limited to pyramid target alleles at multiple loci because the genetics of tolerance in tepary is less studied [24,25,26].

Drought tolerance pathways [27] and QTL are widely studied in common bean [28]. Various candidate genes have been identified [29], most with moderate effects as expected for a complex adaptive trait [26]. Some mechanisms of drought tolerance are controlled through an abscisic acid (ABA) responsive pathway [30]; among which *Asr* (abscisic acid, stress, ripening induced) transcription factors are components [31] of the ABA-dependent pathway [32] that interact with ABA-responsive element promoters [33] for sucrose synthase genes [34]. *Asr1* has been under positive natural selection for drought adaptation in wild common beans from semi-mesic to dry habitats [35] indicating its importance for breeding or gene editing in the future and for the present study.

Meanwhile, other mechanisms of drought tolerance are independent of ABA [30,36]. For instance, drought-responsive element-binding (DREB) protein-encoding *Dreb* genes are also plant-specific, stress-regulated transcription factors that belong to the AP2/EREBP family, but are in the ABA-independent pathway [37,38]. These transcription factors have an AP2 domain and interact with drought-responsive elements (DRE) or promoters found near other genes involved in adaptation to drought [39]. In common bean’s wild genepools, *Dreb2A* exhibited levels of nucleotide diversity above the genomic average, which is indicative of adaptive (divergent) selection across variable habitats differing in natural evapotranspiration and precipitation [40]. In contrast, *Dreb2B* had very low nucleotide diversity relative to neutral reference loci, likely due to purifying selection, so that cultivated accessions have lower diversity than the wild [41].

Upstream of the drought stress response pathways, *ERECTA*-like encoding genes are among the best-characterized transcriptional regulators affecting drought tolerance in plants [42]. These leucine rich repeats (LRR) receptor-like serine/threonine-protein kinase trans-membrane proteins perceive the drought stress signal across the cell membranes where they are located [43]. Specifically, *ERECTA* proteins regulate the frequency and development of stomata on leaves [44], among many other biological processes including development, pathogen defense and phyto-hormone perception [45]. Similarly to *Asr* and *Dreb* genes, an *ERECTA*-encoding gene was associated with ecological differences in common bean wild accessions found across a range of wet to dry habitats. 

In a trait typically regarded as polygenic like drought tolerance [46], these genes are candidates for adaptive introgression [47], as a way to overcome the genetic erosion from the domestication bottlenecks in a cultivated genepool of tepary bean [48]. Since these regulators display signatures of adaptation in wild common bean from habitats with different water regimens, we hypothesized that tepary may exhibit diversity signals at these same genes indicative of adaptation to dry environments. 

With the overall objective of understanding drought tolerance genes in tepary bean, our goals in this study were (1) to estimate drought tolerance in the hybridizing tepary bean clade *s.l.* (*P. acutifolius*–*parvifolius*) using geo-referenced germplasm accessions and associated climate information, and (2) to examine genetic correlations between estimated drought stress in tepary bean and its allele diversity at *Asr2*, *Dreb2B*, and *ERECTA*-encoding genes, which we had previously studied and found to be significantly associated with domestication and drought tolerance in common bean. This will allow the unlocking of drought-related genetic variation hidden in tepary beans and their wild relatives [11,49,50], extending from there into early landraces that together with variation from common bean wild relatives can be used to increase the rate of genetic gain for drought tolerance via inter-specific hybridization, marker-assisted backcrossing [51,52], genomic editing [53], or predictive breeding [54] in any of the *Phaseolus* cultigens.

## 2. Materials and Methods

### 2.1. Plant Material

A panel of diverse tepary bean genotypes representing the allelic variation in the *P. acutifolius*–*parvifolius* clade were considered in this study (23 wild *P. acutifolius*, 6 cultivated *P. acutifolius*, 4 *P. acutifolius* var. *tenuifolius*, and 19 *P. parvifolius*, Figure S1, Table S1). Wild accessions were prioritized over cultivated ones because many cultivars of tepary bean are duplicate or highly similar as indicated by Muñoz, Duque, Debouck, and Blair [16] and Blair, Pantoja, and Carmenza Munoz [9], using AFLP and SSR marker datasets, respectively. Besides, the hybridizing nature of the cultivated-wild tepary bean clade *s.l.* (*P. acutifolius*–*parvifolius*) implied that the effective size of this sampling was higher in terms of available standing adaptive variation and contrasted abiotic responses, allowing for a candidate gene design, as described below. Additional sequencing control genotypes were made up of the common bean accessions BAT93, BAT477, BAT881, and G19833. Seed material and recommendations were provided by D. Debouck and O. Toro of the Genetic Resource Unit and the Food and Agriculture Organization (FAO) Genebank collection, CIAT (http://isa.ciat.cgiar.org/urg/main.do, 10 April 2021).

### 2.2. Habitat-Based Drought Stress Indices

Available geo-referencing of the collection site for each accession was used to extract climate information at a 2.5-min resolution from WorldClim (http://www.worldclim.org, 10 April 2021) using the *dismo* and *raster* packages of the software package R v.4.0.2 (R Core Team). Historical temperature and precipitation values were obtained as monthly averages from 1970 to 2000 in order to estimate habitat Drought Index (DI) ratio (Table S2) using Thornthwaite’s potential classic evapotranspiration (*PET*) model [55]. *PET_j_* computation followed Equation (1) for the ‘*j*’ month, as validated by Cortés, Monserrate, Ramírez-Villegas, Madriñán, and Blair [40], and López-Hernández and Cortés [56], in a diverse panel of common bean accessions.

(1) $${PET}_{j}=\{\begin{array}{ll} 1.6L_{j}{\left(\frac{{10T}_{j}}{I}\right)}^{a} & T_{j}>0\\ 0 & T_{j}\leq 0 \end{array}$$

This *PET_j_* estimate considered explicit temperature effects (*T_j_*, monthly mean air temperature in °C), as well as indirect temperature-related properties via annual heat index (*I*, Equation (2)) and a cubic function of *I* (*a*, Equation (3)).

(2) $$I=\{\begin{array}{ll} \sum_{i=1}^{12}{\left(\frac{T_{j}}{5}\right)}^{1.514} & T_{j}>0\\ 0 & T_{j}\leq 0 \end{array}$$

(3) $$a=6.75*10^{-7}*I^{3}-7.71*10^{-5}*I^{2}+1.792*10^{-2}*I+0.49239$$

The *PET_j_* score in Equation (1) did not just account for temperature drivers, but also incorporated latitudinal adjusted sunlight radiation (*L_j_*, Equation (4)) as a function of day length duration (*D_j_*) and latitude in sexagesimal degrees (Equation (5)).

(4) $$L_{j}=\frac{D_{j}}{12}$$

(5) $$D_{j}=24-\left(\frac{24}{\pi }\right)*{cos}^{-1}\left(\frac{sin\left(\frac{0.8333*\pi }{180}\right)+sin\left(\frac{Latitude*\pi }{180}\right)*{sinA}_{j}}{cos\left(\frac{Latitude*\pi }{180}\right)*{cosA}_{j}}\right)$$

Day length duration (*D_j_*) was in turn corrected for day of year (*J_i_*, day 15 of the ‘*j*’ month), as in Equation (6)).

(6) $$A_{j}={sin}^{-1}\left(0.39795*cos(0.2163108+2*{tan}^{-1}\left(0.9671396*tan\left(0.00860*\left(J_{i}-186\right)\right)\right)\right))$$

Finally, after computing *PET_j_*, monthly Drought Indices (*DI_j_*) were obtained by comparing *PET_j_* estimators with monthly precipitation values (*P_j_*), so that *DI_j_* followed Equation (7) for the ‘*j*’ month, where *D_j_* ≤ 100.

(7) $${DI}_{j}=100*\left(\frac{{PET}_{j}-P_{j}}{{PET}_{j}}\right)$$

The *PET_j_* (Equation (1)) and *DI_j_* (Equation (7)) scores were considered over three, six, and 12 month intervals in the first place, then in an alternative analysis, where the estimations were carried out over four trimesters, with the aim to match tepary bean phenology at any time period over the year with drought stress indicators such as the ones described. 

**Figure 1:**
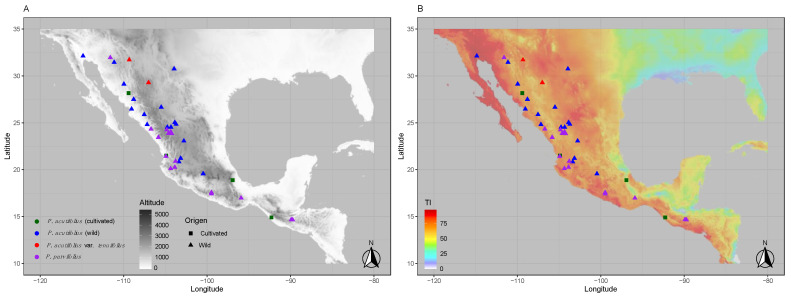
Geographical representation of collection sites for tepary beans *s.l.* (*P. acutifolius–parvifolius* clade) evaluated in this study with (**A**) altitude in meters above sea level as a background and (**B**) drought index on a 30 s grid for the region between the Southern United States and Central America, including the collection hotspot of Northwest Mexico. Dots are colored by taxonomic origin (Table S1) as follows: Green and blue for cultivated and wild *P. acutifolius*, red for wild *P. acutifolius* var. *tenuifolius*, and purple for wild *P. parvifolius*. Altitudes represented in various tones of achromatic gray. Drought severity index based on Thornthwaite’s potential evapotranspiration (PET) model (DI—Thornthwaite’s index) indicated by scales of red (most intense), orange, yellow to green, and blue (least intense). Latitude and longitude represented by grids in both panels. Wild and cultivated accessions marked as filled triangles (▲) and squares (■), respectively.

The timeframes considered accumulated (three, six, and 12 month periods) and non-accumulated (trimesters one, two, three, and four) drought events over seasonal or yearlong time frames in the natural habitat where each accession was collected. A map of collection sites was drawn for the study area at 30 s resolution in R v.4.0.2 using *ggmap* package (Figure 1). This geographical representation considered altitude in one panel and DI in the other showing each tepary bean collections site in parallel. The maps were useful to understand the overall DI, where red areas had higher stress values compared to those graphed in blue with lower drought stress and greater water availability, in comparison to altitude.

### 2.3. DNA Extraction, Candidate Gene Amplification and Sequencing

Total DNA was extracted from young leaves after seedling germination of each tepary bean genotype following the method of Dellaporta et al. [57]. Combinations of primers (Table S3) and amplification conditions of introns and flanking variable sections from three prioritized candidate genes followed standardizations carried out by Cortés, Chavarro, Madriñán, This, and Blair [35], Cortés, This, Chavarro, Madriñán, and Blair [41], and Blair, Cortés, and This [48] for *Asr2*, *Dreb2B*, and *ERECTA*-encoding genes, respectively. The same primers worked for amplicons’ preparation and sequencing. Amplicons’ quality and sizes were checked on a 1.5% agarose-Tris-Borate-EDTA gel containing GelRed (Biotium, Fremont, CA, USA). Successful amplicons with bright bands in the gels were purified using Exo-Sap clean-up reactions in order to be used as templates for subsequent paired-end Sanger sequencing reactions using BigDye Terminator v3.1 Cycle Sequencing Kit (Applied Biosystems, Waltham, MA, USA). Purified samples of the cleaned-up bands were aliquoted to a standard 200 ng of DNA, lyophilized, and sent to Macrogen USA (Psomagen Inc., Rockville, MD, USA) to be run on an ABI prism 3730 automated sequencer with original primers for sequencing and Big Dye chemistry (ABI, Foster City, CA, USA). Four common bean (*P. vulgaris*) genotypes (BAT93, BAT477, BAT881, and G19833) were included as sequencing controls to assist allele calling. 

### 2.4. Patterns of Nucleotide Diversity and Environmental Correlations at Candidate Genes 

Nucleotide alignments were carried out on *Geneious* v4.0 software (Biomatters Ltd., Auckland, New Zealand). The sequences were manually examined for quality of the alignment and single nucleotide polymorphism (SNP) call. Genetic diversity was explored per gene by computing PCA plots in *DnaSP* v5.10 [58], and Neighbor Joining (NJ) dendrograms with 1000 bootstrap iterations in *Mega* v4 [59]. The former software was also used to compute summary statistics of the site frequency spectrum. Pairwise *F_ST_* and median joining haplotype networks for each gene were calculated and drawn (*F_ST_* ranges were plotted per quartiles) in R packages *poppr* and *pegas—ape*, respectively (R Core Team).

Two strategic analyses were performed to identify the correlation between SNPs and environmental conditions of specific collection sites. Firstly, timeframes at three, six, and 12 months were considered. Secondly, timeframes included four trimesters along the year. The environmental indices were then correlated with all timeframes analyzed for the three genes. Environmental correlations with candidate genes implemented GLM, MLM, and CMLM models in *Tassel* v5.0 [60] for SNP markers, and MLM models for gene haplotypes in R’s (v.3.4.4, R Core Team) package *nlme* [61]. SNP and haplotype-based mixed models, respectively, accounted for population strata [62] via an IBS kinship matrix as computed in *Tassel* v5.0 [60], as well as the first two principal genetic components consistent with previously generated SNP [23] and SSR [10] data. Since the candidate gene method had a priori bases on gene functionality and violated the random and independence allelic sampling hypotheses, a FDR equivalent *p*-value threshold of 0.01 was considered as in Oord and Sullivan [63].

## 3. Results

### 3.1. Pervasive Environmental and Allelic Diversity for Three Drought Candidate Genes in Tepary Bean s.l.

Drought stress was found to be extensive in tepary beans based on the climate data for the habitats where they were collected (Figure 1). Wild tepary beans were from the foothills of the dry Sonoran desert mountains of northern Mexico with cultivars collected further afield as far south as Guatemala. 

When observing the map below, we see that *P acutifolius—parvifolius* clade *s.l.* showed a correspondence to dry environments, with *P. acutifolius* and *P. parvifolius* prevalent in desert habitats at various altitudes. Distribution of the var. *tenuifolius* was in the northern area of the range, while wild var. *acutifolius* was from across the full range from Northern to Central Mexico. *P. parvifolius* was distributed in the middle part of the range with a few outliers from both extremes. The source of *P. vulgaris* were not illustrated here because they were not used in the climate analyses. 

**Figure 2:**
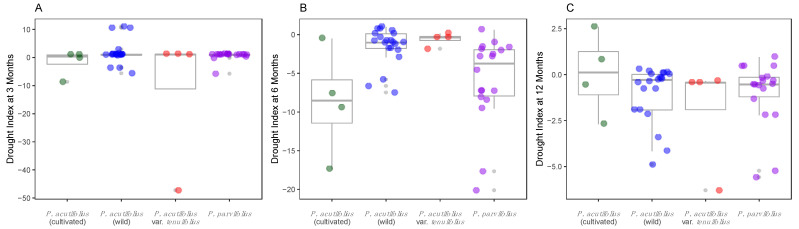
Dispersal graph boxplots for habitat drought stress index in four taxonomical divisions of broad sense (*s.l.*) tepary bean (from the *P. acutifolius–parvifolius* clade) based on the Thornthwaite’s potential evapotranspiration (PET) model. Drought indices computed at (**A**) three, (**B**) six, and (**C**) 12 months. Colors mark taxonomy: Green and blue for cultivated *P. acutifolius* and wild *P. acutifolius*, red for wild *P. acutifolius* var. *tenuifolius*, and purple for wild *P. parvifolius*.

Following mapping and in order to study the variability of the drought stress indices based on taxonomic origin, we performed ANOVA and Tukey tests for the indices with normal behavior, and the pairwise Wilcox test for the non-normal indices corrected by Bonferroni (Figure 2).

Scaled habitat drought stress indices were equivalent for different timeframes, as shown in Figure 2 with boxplots for the three (median = 0.14, CI 95%: 0.13–1.36), six (median = 0.40, CI 95%: −0.66–0.90), and 12 month (median = 0.33, CI 95%:−0.33–1.00); similarly, trimester timeframes analysis showed extensive drought stress, being trimester one (mean = 0.128, CI 95%: −0.63–0.89), trimester two (mean = 2.147, CI 95%: −4.43–8.72), trimester three (mean = −8.65 CI 95%: −13.97–3.32), and trimester four (mean = −0.79 CI 95%: −1.53–0.06) equivalent values for dry habitats especially in the last semester of the year (Figure 2A–C).

### 3.2. Allele Variants at Dreb2B and ERECTA-Encoding Candidate Genes Were Correlated with Drought Stress

Single nucleotide polymorphism alleles and frequencies along with expected heterozygosity (*H_e_*) are found in Table 1, with 13 SNPs in *Asr2*, 8 in *Dreb2B*, and 22 in *ERECTA*. The difference in SNP number for each gene was probably related to the length of the fragment analyzed by sequencing or the nature of polymorphism in the genes. 

**Figure 3:**
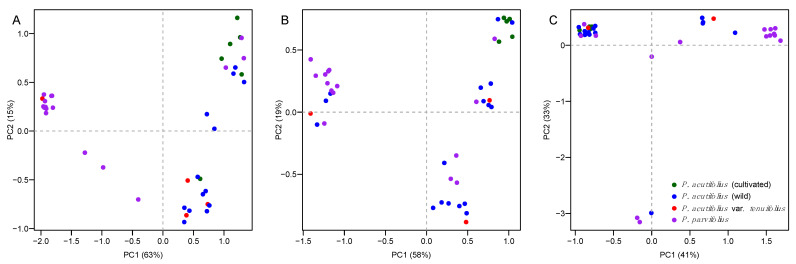
Principal component analyses (PCAs) of genetic polymorphism at (**A**) *Asr2*, (**B**) *Dreb2B*, and (**C**) *ERECTA*-encoding candidate genes for drought tolerance in tepary bean *s.l.* (*P. acutifolius*–*parvifolius* clade). Colors follow Figure 1, by taxonomic origin (Table S1): Green and blue for cultivated and wild *P. acutifolius*, red for *P. acutifolius* var. *tenuifolius*, and purple for *P. parvifolius*, latter two wild entries. First two principal genetic components allow comparisons with previously generated SNP [23] and SSR [10] data.

Grouping of accessions by PCA clustering was minimal based on overall genetic polymorphism at *Asr2* (Figure 3A), *Dreb2B* (Figure 3B), and *ERECTA*-encoding (Figure 3C), respectively. Reconstruction of Neighbor Joining (NJ) dendrograms supported this observation (Figures S2–S4, respectively for the same genes). Genotypes of different taxonomic origins for drought tolerance were generally intermingled (Table S4). More concretely, cultivated *P. acutifolius* was not necessarily more clustered than wild *P. acutifolius* (i.e., *Asr2*, Figure 3A), and these were not assembled separately from *P. acutifolius* var. *tenuifolius* and *P. parvifolius*.

Summary statistics calculated for the site frequency spectrum for each candidate gene in tepary bean *s.l.* revealed contrasting demographic/selection patterns (Table 2). In this analysis, *Asr2* matched the expectations of a semi-structured pairwise mismatch distribution (positive *Tajima’s D* value of 0.873). Meanwhile, for *Dreb2B* and *ERECTA*-encoding genes, signatures of directional/purifying selection (negative *Tajima’s D* values of −0.814 and −0.974, correspondingly) were observed, likely in favor of adaptive alleles selectively advantageous.

**Figure 4:**
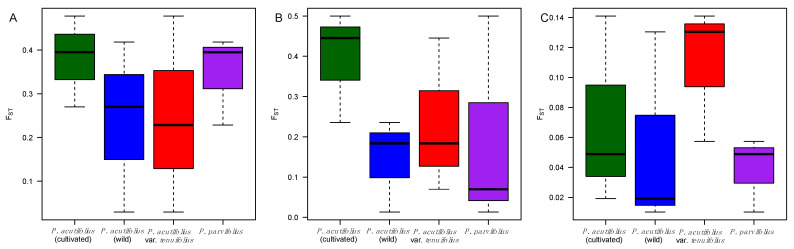
Boxplots of the pairwise F_ST_ distributions at (**A**) *Asr2*, (**B**) *Dreb2B*, and (**C**) *ERECTA*-encoding candidate genes for drought tolerance in tepary bean *s.l.* (*P. acutifolius*–*parvifolius* clade). Boxplots colored by taxonomy: Green and blue for cultivated and wild *P. acutifolius*, red for *P. acutifolius* var. *tenuifolius*, and purple for *P. parvifolius*, the latter two wild.

In a different analysis, boxplots of the pairwise *F_ST_* values were prepared (Figure 4) for each candidate gene across the taxonomic origins to search for significant differences between *Asr2*, *Dreb2B*, and *ERECTA*. Differences were not observed for the first and last of these genes, but a cultivated vs. wild *P. acutifolius* difference was observed for *Dreb2B*.

**Figure 5:**
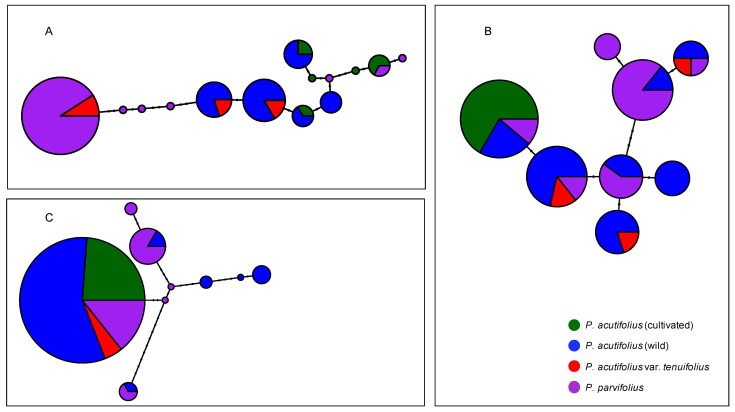
Haplotype networks of genetic polymorphism at (**A**) *Asr2*, (**B**) *Dreb2B*, and (**C**) *ERECTA*-encoding candidate genes for drought tolerance in tepary bean *s.l.* (*P. acutifolius*–*parvifolius* clade). Nodes represent haplotypes, its size relative to its frequency. Marks above each segment are substitutions. Nodes are colored by taxonomy (Table S1): Green and blue for cultivated and wild *P. acutifolius*, red for *P. acutifolius* var. *tenuifolius*, and purple for *P. parvifolius*, the latter two wild accessions.

Haplotype network reconstructions were made for *Asr2* (Figure 5A), *Dreb2B* (Figure 5B), and *ERECTA*-encoding (Figure 5C) candidate genes. For the latter, all cultivated tepary beans shared a single haplotype, while the wild tepary beans from within *P. acutifolius* and from *P. parvifolius* were in various nodes of the networks.

In the test of habitat drought stress correlation with alleles at the three genes, results were consistent across several model types, yet CMLM was stricter than GLM and MLM (Table 3). For instance, GLM showed most of the correlations at *Dreb2B*, being 87.5% of the segregating sites correlated under this model for both timeframes. At *ERECTA*, the correlated SNPs accounted for 27%. MLM, on the other hand, was less prevalent at any timeframe, and CMLM showed no significance to any SNP, likely due to an over-correction or inflated type *β* error. Significant associations were lost when carrying out haplotype-based environmental correlations.

## 4. Discussion

### 4.1. Tepary Bean s.l. Is a Reservoir of Novel Alleles at Candidate Genes for Drought Tolerance

Tepary bean is unique compared to common bean in being monophyletic, having a single genepool, and furthermore, low introgression from other species due to its highly autogamous inbreeding status. This is reflected in the results of the candidate gene analysis here for wild and cultivated tepary accessions. Genes *Asr2*, *Dreb2B*, and *ERECTA* harbored signatures of directional/purifying selection, in favor of adaptive alleles represented by various SNPs significantly correlated with the environmental drought indices (*p*-value < 0.05 and 0.01). Network analysis also found haplotypes more frequent for wild accessions than cultivars. These results suggest that tepary bean *s.l.*, especially wild species *P. parvifolius* and wild accessions of *P. acutifolius* var. *acutifolius* or var. *tenuifolius,* were reservoirs of novel alleles at candidate genes for tolerance, as expected for a drought-tolerant species that originated in arid environments. Overall diversity is thought to increase consecutively from low levels in cultivated *P. acutifolius*, to intermediate levels in wild *P. acutifolius* var. *acutifolius* or var. *tenuifolius*, to higher levels in *P. parvifolius*. For this reason, sampling of larger amounts of wild accessions was important and representative of the diversity found in previous characterizations [9,16]. 

Allele diversity in *Asr2*, *Dreb2B*, and *ERECTA*-encoding candidate genes was recovered in tepary bean *s.l*. (including all of those in the primary and secondary genepool, namely the variants which are all inter-crossable, and the wild species *P. parvifolius*, which can also be crossed with no barrier to *P. acutifolius*). This is appealing because variation of cultivated tepary bean is limited by strong genetic bottlenecks, given the paucity of diversity in seed color or SSR marker genotypes in cultivated accessions [9].

The observation above of diversity signals indicative of adaptation to drought at these candidate genes brings us back to the hypothesis for this research and questions of whether wild tepary beans and *P. acutifolius* var. *tenuifolius* and *P. parvifolius* are more variable for drought gene alleles than cultivated types. Although we cannot answer the question definitively, it appears that drought tolerance candidate genes were not as severely affected by domestication as suggested by the limited allele diversity found for SSR markers or few seed colors in the crop [48]. The main reason for this might be that drought tolerance was likely an “untouched” trait during the domestication of tepary bean, being a drought-tolerant species complex from very arid environments. Increases in functional diversity—but not in expression diversity [67], had already been noticed at target traits during the domestication of common bean as revealed by genome-wide enrichment of non-synonymous substitutions [68], and may also be responsible for adaptive functions, beyond drought tolerance, in tepary bean.

Analyses of the same candidate genes for drought tolerance reveal contrasting patterns in the closely related common bean species. Signatures of directional and divergent selection are observed in *Asr1* [35] and *Dreb2A* [40] for genes in wild accessions from semi-mesic to dry habitats, and an *ERECTA*-encoding gene exhibits haplotype correlations with ecological differences in diverse common beans [48]. Additionally, when these candidate genes were screened in cultivated genepools and races of common bean, diversity signals indicative of adaptation to drought are in line with expectations concerning selection during the domestication syndrome or simply reflect demographic bottlenecks.

### 4.2. Unlocking Useful Genetic Variation for Drought Tolerance

Since genetic variation at drought candidate genes is partly recovered for tepary bean, this polymorphism likely predates the domestication event. Still, wild tepary bean, especially from *P. acutifolius* var. *acutifolius* and var. *tenuifolius* with no genetic barriers to the cultivated types, might be useful to add new variability for cultivars. *P. parvifolius* has also been shown to be not too distant from tepary beans [9] and crossability should be tested with multiple accessions. The domestication of tepary bean in the xeric region of the Sonoran Desert, along mountain arroyos and up to the Mexico–USA border could have reinforced allele variation at drought genes in favor of tolerant phenotypes. This appears to hold true for both var. *tenuifolius* and var. *acutifolius* in the wild accessions. Inter-crossing would be useful with a panel of diverse, multi-colored tepary beans with multiple seed sizes since most cultivars are white seeded [17], but some cream, yellow and tan to brown colored cultivated accessions exist [16]. Among our wild accessions, most had the typical wild tepary bean patterned seed, which are mottled, small in size, angular in shape, and gray to black in seed color.

Drought tolerance has traditionally not been a trait under direct selection as part of the domestication syndromes of *Phaseolus* bean species [12]. Seed germination, color and size, or pod size and dehiscence plus flowering time [69], *Rhizobia* symbiosis [70,71], secondary metabolites [72], and circadian clock components [73] are all functions more commonly regulated during the domestication process of legumes. Still, the nature of the strength and the direction of the selection have varied across domestications in *Phaseolus*. This is the case for common bean with two or three domestication events [74], and lima bean also with multiple possible domestications [75]. 

However, since tepary bean is a drought-tolerant species that originated in drought-prone environments only once, drought tolerance may have preceded the domestication syndrome, and could have been a trait unaffected by domestication despite strong population bottlenecks of a single domestication of light colored seeded types [9]. Unlocking newer genetic variation for drought tolerance in tepary bean can harness further resequencing of allelic diversity and marker validation in a broader basis of germplasm material, wild accessions, and related species, within the limitations of the few accessions collected in gene banks.

Returning to the allele diversity found among *Phaseolus*, abiotic stress responsive candidate genes exhibit comparable patterns of diversity when contrasting orthologous sequences across species. For instance, the common bean’s *Asr2* matches the bimodal expectations of a pairwise folded site frequency spectrum [35] inferred using neutral reference loci [76], while *Dreb2B* shows signatures of purifying selection [40]. This contrasts with the double domestication of common bean [77]. *Dreb2B* and *ERECTA*-encoding orthologous genes display analogous selective signatures in tepary bean *s.l.*, as supported by *Tajima’s D* summary statistic, despite *P. vulgaris* and *P. acutifolius*–*parvifolius* not being sister clades [12,78]. Parallel studies might be done in *P. lunatus*, an outlier among the *Phaseolus* or in related species as Mesoamerican *P. vulgaris*, *P. dumosus* (year-long), and *P. coccineus* (scarlet runner bean).

Furthermore, full sequencing of linked and well-understood genic regions, e.g., [79], as compared to “random” discovery of SNP markers in linkage equilibrium [80,81], allows for a more precise application of analytical tools, targeting adaptation in wild [82] and semi-domesticated materials [83]. Techniques such as gene-based species tree reconstruction [84] and inferences of the mutation/selection balance [85,86] presuppose physical linkage among markers. As perspective for oncoming studies, we recommend leveraging [87] tools capable of discerning among genuine signatures of adaptive selection to drought from those due to spurious effects [88] related to the demography of the domestication bottlenecks [69,89,90]. Phylogeographic inferences, e.g., [91] will also be improved by assuming independent gene mutation models [92], yet unlinked marker inferences are already available [93,94,95]. Finally, harnessing polygenic adaptive scores from gene-based and genome-wide prediction models [96,97,98] will help building a more cohesive picture of natural drought adaptation and vulnerability [99] in the face of climate change [100], with a focus on highly heterogeneous mountain geographies [101]. 

As the candidate gene approach bypasses the theoretical limitations of using biallelic genetic markers, it is still an efficient and cost-effective methodological alternative, e.g., [102], especially at advanced stages in the genetic mapping of key traits or for marker validation; and in “orphan” species short either on funding or genetic knowledge base. The three genes we analyzed showed significant SNPs (*p*-value < 0.05 and 0.01, the latter a FDR equivalent [62] for a candidate gene [63] design) given habitat drought stress DI (at three, six, and 12 months) in one, five, and four segregating sites for *Asr2*, *Dreb2B*, and *ERECTA*-encoding genes, respectively considering semester timeframes as well, supporting the hypothesis that the genes are key for drought tolerance in tepary beans. The utility of genotype x environment adaptation variables based on geo-references collection sites for the study of candidate genes in wild and cultivated accessions is demonstrated by our study.

For well-studied traits with relatively conserved pathways, such as drought tolerance, the candidate gene approach is still informative despite de novo high-throughput genome-wide genotyping [103,104,105]. While reduced genome complexity genotyping techniques, like genotyping-by-sequencing [106], perform well in medium-size panels, the candidate gene approach allows deeper genotyping of fewer gene regions [107,108] for traits influenced by variants with intermediate effects, e.g., [109,110].

## 5. Conclusions

In summary, our candidate gene study allowed us to delve into the signatures of directional/purifying selection, in favor of adaptive alleles, or the frequency of haplotypes among taxonomic groups or correlated with the environmental drought indices. The results suggested that tepary bean *s.l.*, especially wild accessions, could be sources of novel alleles for breeding of further drought tolerance in the cultivated accessions of an already drought-tolerant orphan crop. Cultivated tepary bean, being low in diversity [9], would benefit from wide crosses with wild relatives to obtain new traits such as new leaf shapes and new seed colors while maintaining the drought tolerance that it innately possesses.

## Figures and Tables

**Table 1:** SNP marker polymorphisms at *Asr2*, *Dreb2B*, and *ERECTA*-encoding drought candidate genes in tepary bean *s.l.* SNPs in coding regions are marked in bold, and non-synonymous variants are in bold and underlined.

Gene Name	Position (bp)	Major Allele	Major Allele Frequency	Minor Allele	Minor Allele Frequency	*H_e_*
*Asr2*	1	C	0.5	T	0.5	0.5
***Asr2***	**101**	**A**	**0.67**	**G**	**0.33**	**0.44**
*Asr2*	185	T	0.73	A	0.27	0.4
*Asr2*	188	T	0.55	A	0.45	0.5
*Asr2*	189	A	0.67	T	0.33	0.44
*Asr2*	190	A	0.74	T	0.26	0.39
*Asr2*	191	T	0.69	A	0.31	0.43
*Asr2*	192	T	0.69	A	0.31	0.43
*Asr2*	200	A	0.88	T	0.12	0.21
*Asr2*	246	T	0.88	A	0.12	0.21
***Asr2***	**407**	**G**	**0.67**	**A**	**0.33**	**0.44**
***Asr2***	**455**	**C**	**0.74**	**A**	**0.26**	**0.39**
***Asr2***	**486**	**C**	**0.9**	**T**	**0.1**	**0.17**
***Dreb2B***	**24**	**C**	**0.89**	**G**	**0.11**	**0.2**
***Dreb2B***	**33**	**C**	**0.64**	**G**	**0.36**	**0.46**
***Dreb2B***	**81**	**A**	**0.93**	**T**	**0.07**	**0.13**
***Dreb2B***	**134**	**A**	**0.68**	**G**	**0.32**	**0.43**
***Dreb2B***	**135**	**C**	**0.68**	**T**	**0.32**	**0.43**
***Dreb2B***	**136**	**A**	**0.68**	**G**	**0.32**	**0.43**
***Dreb2B***	**342**	**C**	**0.8**	**T**	**0.2**	**0.33**
***Dreb2B***	**357**	**G**	**0.82**	**C**	**0.18**	**0.3**
*ERECTA*	32	C	0.85	T	0.15	0.26
*ERECTA*	33	T	0.53	A	0.48	0.5
*ERECTA*	46	C	0.8	T	0.2	0.32
*ERECTA*	47	A	0.8	G	0.2	0.32
*ERECTA*	55	T	0.93	A	0.08	0.14
***ERECTA***	**137**	**G**	**0.93**	**A**	**0.08**	**0.14**
*ERECTA*	172	G	0.93	A	0.08	0.14
*ERECTA*	187	G	0.95	A	0.05	0.1
*ERECTA*	189	C	0.93	T	0.08	0.14
*ERECTA*	223	T	0.95	C	0.05	0.1
*ERECTA*	228	C	0.93	T	0.08	0.14
*ERECTA*	285	T	0.93	C	0.08	0.14
*ERECTA*	286	G	0.93	A	0.08	0.14
*ERECTA*	311	C	0.63	A	0.38	0.47
*ERECTA*	449	C	0.93	G	0.08	0.14
*ERECTA*	476	T	0.65	A	0.35	0.46
*ERECTA*	480	A	0.93	G	0.08	0.14
***ERECTA***	**615**	**T**	**0.93**	**C**	**0.08**	**0.14**
***ERECTA***	**637**	**G**	**0.9**	**A**	**0.1**	**0.18**
***ERECTA***	**683**	**T**	**0.93**	**A**	**0.08**	**0.14**
***ERECTA***	**725**	**C**	**0.63**	**A**	**0.38**	**0.47**
***ERECTA***	**734**	**T**	**0.95**	**G**	**0.05**	**0.1**

**Table 2:** Summary statistics of the site frequency spectrum at *Asr2*, *Dreb2B*, and *ERECTA*-encoding candidate genes for drought tolerance in tepary bean *s.l.* (*P. acutifolius*–*parvifolius* clade). Depicted summary statistics include ***S*:** Number of polymorphic (segregating) sites (enforced *maf* > 0.05 for the entire dataset), ***maf*:** Average minimum allele frequency, ***H_e_*:** average expected heterozygosity—as a measure of the polymorphism information content (PIC), ***π*:** Nucleotide diversity [64], ***θ_W_*:** Theta of Watterson—per site from *S* [65], and ***Tajima’s D*** [66]. Only variable taxa with enough sampling to make per-population computations reliable are kept.

Gene Name	Taxa	Length (*bp*)	*S*	*maf*	*H_e_*	*π*	*θ_W_*	*Tajima’s D*
*Asr2*	All	547	13	0.29	0.38	0.017	0.014	0.873
*Asr2*	*P. acutifolius* (cultivated)	548	13	0.21	0.33	0.009	0.010	−0.816
*Asr2*	*P. acutifolius* (wild)	548	9	0.23	0.36	0.005	0.005	0.314
*Asr2*	*P. parvifolius*	548	15	0.17	0.29	0.008	0.008	−0.079
*Dreb2B*	All	373	8	0.24	0.34	0.012	0.016	−0.814
*Dreb2B*	*P. acutifolius* (wild)	374	9	0.18	0.29	0.007	0.007	0.128
*Dreb2B*	*P. parvifolius*	374	11	0.16	0.27	0.008	0.009	−0.512
*ERECTA*	All	750	22	0.15	0.22	0.009	0.012	−0.974
*ERECTA*	*P. acutifolius* (cultivated)	751	8	0.25	0.38	0.005	0.005	0.142
*ERECTA*	*P. acutifolius* (wild)	751	22	0.10	0.18	0.005	0.009	−1.519
*ERECTA*	*P. parvifolius*	751	25	0.18	0.30	0.009	0.010	−0.447

**Table 3:** Habitat drought stress significant correlations with SNP polymorphisms at *Asr2*, *Dreb2B*, and *ERECTA*-encoding candidate genes for drought tolerance in tepary bean *s.l.* (*P. acutifolius*–*parvifolius* clade). Habitat drought stress (Table S2) was computed as a drought index (*DI*) using Thornthwaite’s evapotranspiration model at three, six, and 12 months as well as trimester timeframes (*T1* to *T4*). Environmental correlations with each candidate gene were determined using generalized, mixed, and compressed mixed (GLM, MLM, and CMLM) linear models accounting for population stratification via an IBS kinship matrix as implemented in Tassel v5.0. Only significant associations are depicted; their *p*-value estimates are bolded (*p*-value < 0.05) and underlined (*p*-value ≤ 0.01), the latter a FDR equivalent for a candidate gene design [63]. SNPs in coding regions are in bold, and non-synonymous underlined.

SNP		GLM *p*-Value			MLM *p*-Value			GLM *p*-Value				MLM *p*-Value			
Gene	*bp*	*3 m*	*6 m*	*12 m*	*3 m*	*6 m*	*12 m*	*T 1*	*T 2*	*T 3*	*T 4*	*T 1*	*T 2*	*T 3*	*T 4*
*Asr*	1	0.421	**0.050**	0.183	0.594	0.456	0.751	0.467	0.066	0.969	**0.033**	0.594	0.422	0.505	0.094
***Dreb2B***	**81**	0.450	0.921	0.180	0.893	0.810	**0.050**	0.451	0.852	**0.002**	0.144	0.893	0.762	**0.009**	0.487
***Dreb2B***	**134**	0.379	0.672	0.100	0.297	0.465	0.400	0.380	0.597	**0.026**	0.144	0.297	0.488	0.737	0.464
***Dreb2B***	**135**	0.379	0.672	0.100	0.297	0.465	0.400	0.380	0.597	**0.026**	0.144	0.297	0.488	0.737	0.464
***Dreb2B***	**136**	0.379	0.672	0.100	0.297	0.465	0.400	0.380	0.597	**0.026**	0.144	0.297	0.488	0.737	0.464
***Dreb2B***	**342**	0.134	0.162	0.064	0.276	**0.048**	0.219	0.135	0.215	0.729	0.504	0.276	0.054	0.851	0.234
*ERECTA*	32	0.384	**0.010**	0.342	0.274	**0.040**	0.530	0.384	**0.010**	0.654	0.923	0.274	0.077	0.943	0.922
*ERECTA*	46	0.519	**0.009**	0.145	0.891	0.202	0.089	0.520	**0.005**	0.344	0.768	0.891	0.410	0.122	0.765
*ERECTA*	47	0.519	**0.009**	0.145	0.891	0.202	0.089	0.520	**0.005**	0.344	0.768	0.891	0.410	0.122	0.765
*ERECTA*	187	0.891	**0.001**	0.449	0.984	**0.010**	0.556	0.892	**0.001**	0.891	0.885	0.984	**0.012**	0.947	0.883

## Data Availability

Data analysis scripts and datasets are archived at Dryad Digital Repository with DOI https://doi.org/10.5061/dryad.bg79cnp8j (11 August 2020).

## Supplementary Materials

The following are available online at https://www.mdpi.com/article/10.3390/genes12040556/s1; Figure S1: Tepary bean accessions georeferenced; Figures S2–S4: Neighbor Joining (NJ) dendrograms with 1000-iterations bootstrap support, respectively, for *Asr2*, *Dreb2B* (both under Tamura’s 3-parameter, *T92*, substitution model with a *γ* distribution), and *ERECTA* (under Jukes–Cantor evolution substitution model) candidate genes; Table S1: Tepary bean passport data; Table S2: Habitat drought stress estimates in Tepary bean; Table S3: Primer combination; Table S4: Principal Components for each Tepary bean accession.
