# Supplementary figures and images for: Allelic Diversity at Abiotic Stress Responsive Genes in Relationship to Ecological Drought Indices for Cultivated Tepary Bean, Phaseolus acutifolius A. Gray, and Its Wild Relatives
Source: Genes (Basel). 2021 Apr 12;12(4):556. doi: 10.3390/genes12040556 (PMC8070098; doi:10.3390/genes12040556)

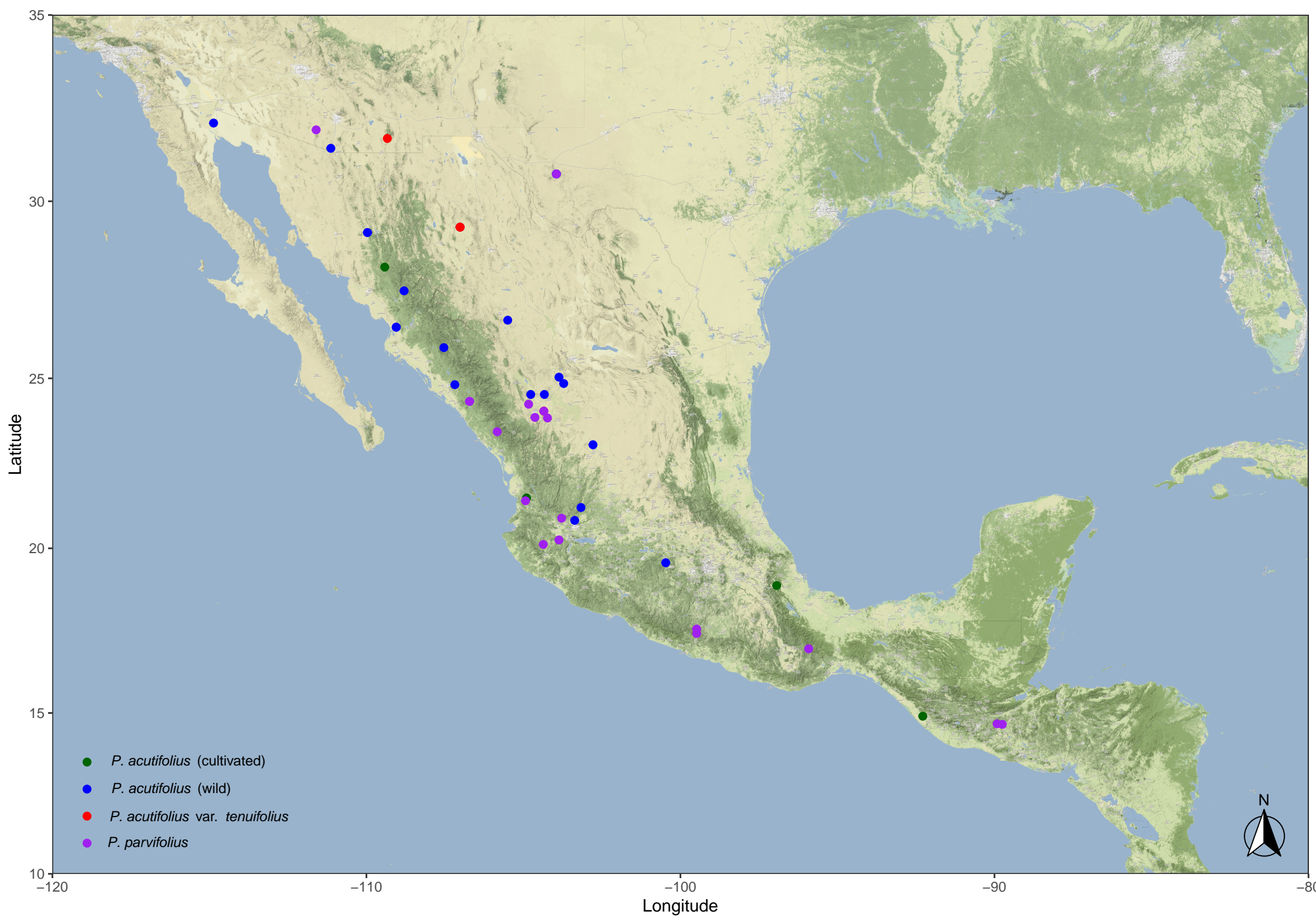

Supplement: Supplementary file 1 [file genes-12-00556-s001.zip › genes-1154598-supplementary/Figure_S1.pdf]

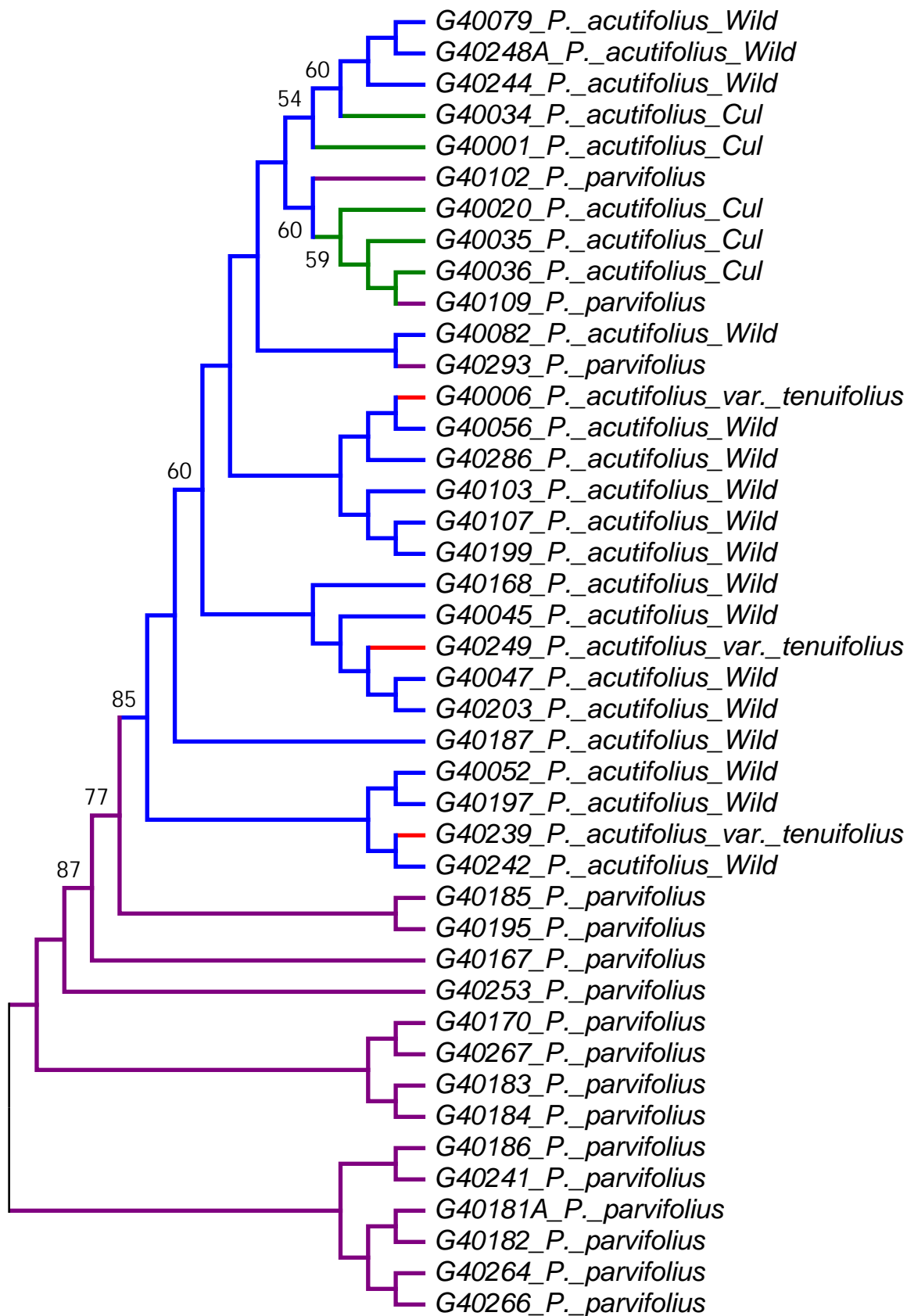

Supplement: Supplementary file 1 [file genes-12-00556-s001.zip › genes-1154598-supplementary/Figure_S2_ASR-NJ.pdf]

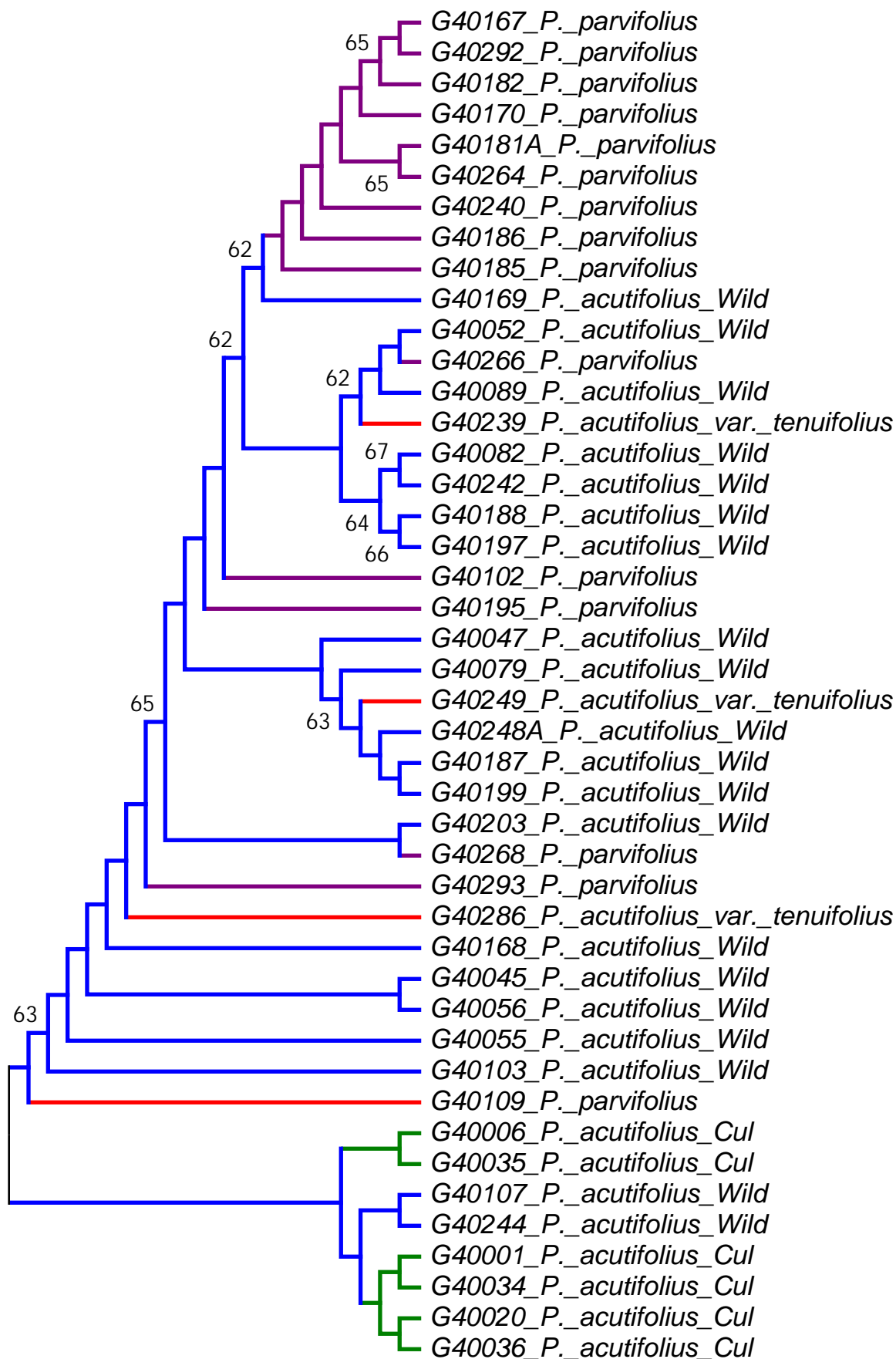

Supplement: Supplementary file 1 [file genes-12-00556-s001.zip › genes-1154598-supplementary/Figure_S3_DREB-NJ.pdf]

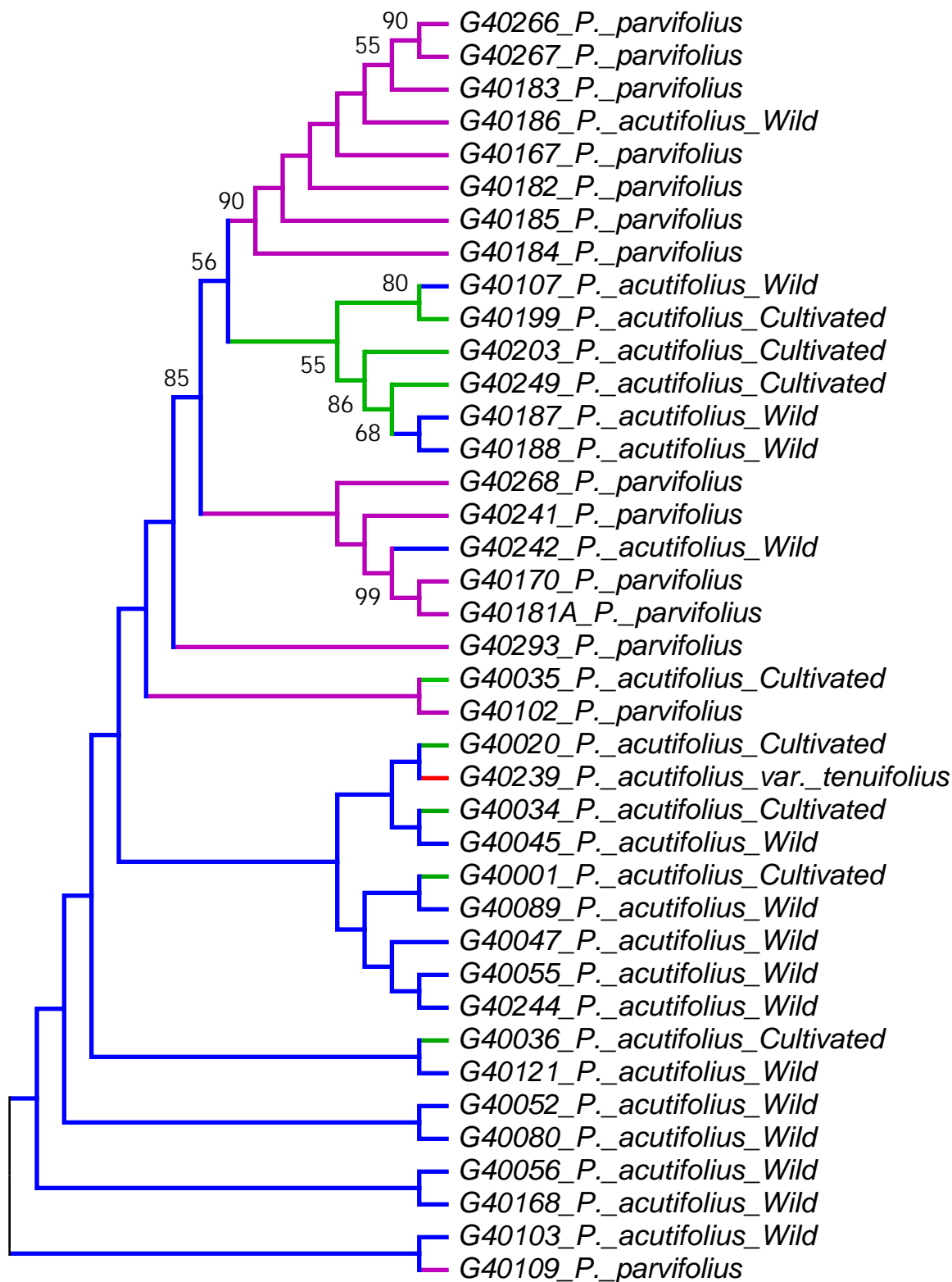

Supplement: Supplementary file 1 [file genes-12-00556-s001.zip › genes-1154598-supplementary/Figure_S4_ERECTA-NJ.pdf]
